# Spotlight on overlooked lignin monomers: Hydroxycinnamaldehydes

**DOI:** 10.1093/plphys/kiad589

**Published:** 2023-11-02

**Authors:** Dyoni M Oliveira, Dechang Cao

**Affiliations:** Assistant Features Editor, Plant Physiology, American Society of Plant Biologists; Department of Plant Biotechnology and Bioinformatics, Ghent University, 9052 Ghent, Belgium; VIB Center for Plant Systems Biology, 9052 Ghent, Belgium; Assistant Features Editor, Plant Physiology, American Society of Plant Biologists; Germplasm Bank of Wild Species, Kunming Institute of Botany, Chinese Academy of Sciences, Kunming, Yunnan 650201, China

Can you imagine a world without lignin? Before lignin was present in vascular plants approximately 470 million years ago, the ancestors of plants could only survive in aquatic and humid environments (Bowman 2022). The incorporation of lignin into the cell walls of early vascular plants contributed to their ability to grow tall by bestowing them with structural rigidity and long-distance water transportation. Lignin, therefore, has been a game-changer in the evolution of plants. Although the exact total carbon storage in the form of lignin remains unknown, it has been estimated that around 20% of the total carbon fixed by photosynthesis is incorporated into lignin by terrestrial plants (Ruiz-Dueñas and Martínez 2009), which makes lignin the most abundant aromatic biopolymer on Earth.

The canonical monolignols *p*-coumaryl, coniferyl, and sinapyl alcohols are synthesized in the cytoplasm before their export to the apoplast, where they are incorporated by oxidative coupling into lignin as*p*-hydroxyphenyl (H), guaiacyl (G), and syringyl (S) units (Ralph et al. 2019). However, coupling of the lignin dimers and oligomers, as well as perturbation of the lignin biosynthetic pathway, can also result in the over-accumulation of lignin monomers that are typically present in low levels or not at all in the lignins of wild-type plants. There are at least 35 different aromatic monomers of lignin (Vanholme et al. 2019). These so-called lignin monomers harbor substantial chemical diversity that makes lignin an ideal source of new aromatics for biorefining to produce high-value bioproducts (Sun et al. 2018). In this regard, new knowledge about the chemical composition and structure of lignin brings new opportunities toward lignin valorization for a sustainable bioeconomy (Oliveira and Cesarino 2023).

Given that lignin units are primarily connected by ether and carbon-carbon bonds, it is very challenging to selectively break lignin bonds and analyze them using degradative methods. Nuclear magnetic resonance (NMR) methods are extremely powerful nondegradative approaches that allow in-depth exploration of “new” structures in the lignins with sufficient resolution to discern differences in the inter-monomer linkages (Lu and Ralph 2003; Ralph et al. 2019), similar to how a jigsaw is assembled from its pieces. In this issue of *Plant Physiology*, Yoshioka et al. (2023) use a combination of chemical assays, 2D NMR, and metabolomics to build on their previous findings and demonstrate the existence of hydroxycinnamaldehyde-derived benzofuran components and their coupling modes during lignification.

The starting point for the work of Yoshioka et al. (2023) was the previously observed presence of non-elucidated aldehyde signals in the 2D NMR structural profile of a natural mulberry (*Morus alba*) mutant (Yamamoto et al. 2020). Being distinct from common woods, the mulberry mutant has unusual red-color xylem tissues, which was demonstrated to result from a natural mutation of a gene encoding the cinnamyl alcohol dehydrogenase (CAD) (Yamamoto et al. 2020). Loss-of-function or down-regulation of *CAD* genes has been reported to lead to strong incorporation of hydroxycinnamaldehydes into lignins, reduction in lignin content, and the distinctive reddish wood (Van Acker et al. 2017).

Hydroxycinnamaldehydes, sinapaldehyde and coniferaldehyde, as well as the hydroxybenzaldehydes, originate from the truncation of the monolignol pathway. It has been proposed that hydroxycinnamaldehydes are integrally incorporated into lignin, either as end-groups by the minor oxidation of monolignols in the apoplast or as parts of the polymer by the incorporation of cytosolic hydroxycinnamaldehyde monomers (Ralph et al. 2023). CAD is the enzyme involved in the last step of monolignol biosynthesis, reducing hydroxycinnamaldehydes into their corresponding hydroxycinnamyl alcohols, the monolignols. There were obvious but unresolved aldehyde signals in the 2D HSQC NMR spectra of the CAD-deficient mulberry mutant (Yamamoto et al. 2020), which brings a new opportunity to study detailed coupling modes of these hydroxycinnamaldehydes during lignification.

The authors therefore examined a chemical system based on synthetic low-molecular lignin oligomers with coniferaldehyde and elucidated that the NMR signals belonged to hydroxycinnamaldehyde-derived benzofuran components (Fig. 1). These benzofuran components represent an 8-5 coupling mode of hydroxycinnamaldehyde, being distinct from commonly known hydroxycinnamaldehyde 8-O-4 coupling units of lignin. To explore whether these hydroxycinnamaldehyde-derived benzofuran components are soluble compounds before their incorporation into lignin, Yoshioka et al. turned to targeted metabolomics searching for compounds containing benzofuran moieties in metabolic extracts of wild-type poplar (*Populus tremula* × *alba*) xylem and confirmed the presence of benzofuran-containing compounds. Even higher levels of benzofuran-containing compounds were detected in CAD-deficient poplar xylem. In this way, the authors matched the presence of putative benzofuran-containing metabolites to hydroxycinnamaldehyde-derived benzofuran components incorporated into the lignin polymers. Furthermore, the reevaluation of new analyses facilitated the discoveries and provided evidence for the incorporation of a monomer into lignin.

**Figure 1.**
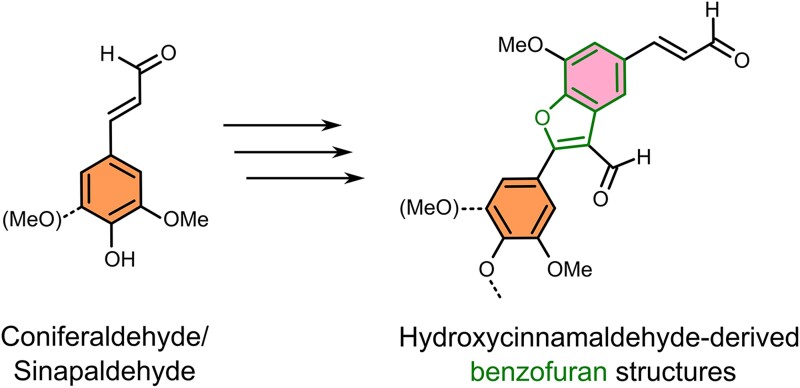
Scheme of the dimerization of coniferaldehyde or sinapaldehyde to form the hydroxycinnamaldehyde-derived benzofuran structures in lignin polymers. The benzofuran structure is indicated in green bonds.

In addition to the well-established hydroxycinnamaldehyde and hydroxybenzaldehyde end-groups in lignins, the authors also examined the aldehyde region of NMR spectra. They screened various lignins and successfully identified hydroxycinnamaldehyde signals not only in CAD-deficient plants like mulberry, poplar, pine (*Pinus taeda*), and *Nicotiana benthamiana* (a close relative of tobacco) but also in wild-type poplar, maize (*Zea mays*), and ryegrass (*Lolium multiflorum*).

These findings revealed “new” components of lignin polymers and shed light on the complex lignin composition and the amazing lignification process. Evaluation of the dynamics of these new lignin components in different developmental stages and cell types would be useful to explore their biological significance. In addition, the 8-5 coupling of hydroxycinnamaldehydes gives rise to phenylcoumaran structures, but the parent phenylcoumarans can hardly be seen whereas the benzofuran products were detected in the lignins investigated here. Disproportionation reactions appear to have priority over radical coupling for the phenolic radicals from such phenylcoumaran compounds. Further studies on the biosynthetic pathways are needed to examine why benzofuran products are preferred over phenylcoumaran structures during the lignification process.

## Dive Curated Terms

The following phenotypic, genotypic, and functional terms are of significance to the work described in this paper:

lignin CHEBI: CHEBI:6457

## References

[kiad589-B1] Bowman JL . The origin of a land flora. Nat Plants. 2022:8(12):1352–1369. 10.1038/s41477-022-01283-y 36550365

[kiad589-B2] Lu F , Ralph J. Non-degradative dissolution and acetylation of ball-milled plant cell walls: high-resolution solution-state NMR. Plant J. 2003:35(4):535–544. 10.1046/j.1365-313X.2003.01817.x 12904215

[kiad589-B3] Oliveira DM , Cesarino I. Genome editing of wood for sustainable pulping. Trends Plant Sci. 2023. 10.1016/j.tplants.2023.10.007 37838517

[kiad589-B4] Ralph J , Kim H, Lu F, Smith RA, Karlen SD, Nuoendagula, Yoshioka K, Eugene A, Liu S, Sener C, et al Lignins and lignification. In: Salminen J-P, Wähälä K, de Freitas V, Quideau S, et al, editors. Recent advances in polyphenol research. West Sussex (UK): John Wiley & Sons, Inc.; 2023. p. 1–50. 10.1002/9781119844792

[kiad589-B5] Ralph J , Lapierre C, Boerjan W. Lignin structure and its engineering. Curr Opin Biotechnol. 2019:56:240–249. 10.1016/j.copbio.2019.02.019 30921563

[kiad589-B6] Ruiz-Dueñas FJ , Martínez ÁT. Microbial degradation of lignin: how a bulky recalcitrant polymer is efficiently recycled in nature and how we can take advantage of this. Microb Biotechnol. 2009:2(2):164–177. 10.1111/j.1751-7915.2008.00078.x 21261911 PMC3815837

[kiad589-B7] Sun Z , Fridrich B, de Santi A, Elangovan S, Barta K. Bright side of lignin depolymerization: toward new platform chemicals. Chem Rev. 2018:118(2):614–678. 10.1021/acs.chemrev.7b00588 29337543 PMC5785760

[kiad589-B8] Van Acker R , Déjardin A, Desmet S, Hoengenaert L, Vanholme R, Morreel K, Laurans F, Kim H, Santoro N, Foster C, et al Different routes for conifer- and sinapaldehyde and higher saccharification upon deficiency in the dehydrogenase CAD1. Plant Physiol. 2017:175(3):1018–1039. 10.1104/pp.17.00834 28878036 PMC5664467

[kiad589-B9] Vanholme R , De Meester B, Ralph J, Boerjan W. Lignin biosynthesis and its integration into metabolism. Curr Opin Biotechnol. 2019:56:230–239. 10.1016/j.copbio.2019.02.018 30913460

[kiad589-B10] Yamamoto M , Tomiyama H, Koyama A, Okuizumi H, Liu S, Vanholme R, Goeminne G, Hirai Y, Shi H, Takata N, et al A century-old mystery unveiled: Sekizaisou is a natural lignin mutant. Plant Physiol. 2020:182(4):1821–1828. 10.1104/pp.19.01467 32051179 PMC7140961

[kiad589-B11] Yoshioka K , Kim H, Lu F, De Ridder N, Vanholme R, Kajita S, Boerjan W, Ralph J. Hydroxycinnamaldehyde-derived benzofuran components in lignins. Plant Physiol. 2024:194(3):1370–1382. 10.1093/plphys/kiad514 37773018

